# Old Doctors or New?

**Published:** 1891-11-28

**Authors:** George Brown

**Affiliations:** 29, Threadneedle Street, E.C.


### Editor's Letter Box.

[Our correspondents are reminded that prolixity of statement is a great bar to publication, and that brevity of style and conciseness of statement greatly facilitate early insertion.]

### OLD DOCTORS OR NEW?

Sir,—Your remarks under the above heading in last Saturday's Hospital would have been very *apropos* had I requested Mr. Wheelhouse and Sir B. W. Foster to retire in favour of myself and Dr. Alderson. Mr. Wheelhouse in his letter to the *British Medical Journal*, on which your article appears to be founded, misquoted me—whether by accident or design it is not for me to say—as may be seen from the enclosed copy of my letter to him, which, in justice to myself, I will thank you to publish.—I am, &c., George Brown.

29, Threadneedle Street, E.C.,
November 17th, 1891.

[Copy.]

29, Threadneedle Street, E.C., October 29th, 1891.

C. G. Wheelhouse, Esq.

Dear Sir,—You will have heard probably ere this that it is my intention to offer myself as a candidate for a seat on the General Medical Council. I am anxious that you should understand that I am influenced to oppose the re-election of yourself and Sir B. W. Foster solely by the feeling that it is the duty of general practitioners to enter a protest against our direct representatives being drawn from the ranks of consultants or those associated with the governing bodies of medical corporations or teaching institutions. The seats for direct representatives were given in response to a demand made on behalf of general practitioners, and I regret that you and Sir B. W. Foster should take advantage of the great influence you wielded through the British Medical Association to prevent those seats being filled by general practitioners. Of course I do not contest your right to offer yourself for re-election. I may, however, say that it would have been a graceful act on the part of Sir B. W. Foster and yourself to have left the field clear for general practitioners.

Assuring you of my high esteem of your personal qualities and general approval of the work you have done since you have been a member of the Council, I remain yours faithfully (Signed) George Brown.

